# Subjective and objective sleep quality does not predict behavioural episodic foresight in younger or older adults

**DOI:** 10.1038/s41598-023-28183-1

**Published:** 2023-01-19

**Authors:** Olivia P. Demichelis, Sarah A. Grainger, Kate T. McKay, Lucy Burr, Joanne Kim, Julie D. Henry

**Affiliations:** 1 School of Psychology, The University of Queensland, Brisbane, Australia; 2 School of Medicine, The University of Queensland, Brisbane, Australia; 3 Department of Respiratory Medicine, Mater Health, Brisbane, Australia

**Keywords:** Psychology, Cognitive ageing, Learning and memory

## Abstract

Episodic foresight refers to one’s capacity to use imagined scenarios to guide future-directed behaviors. It is important in facilitating complex activities of daily living, such as managing finances. Broader literature shows that older adults perform more poorly on tests of episodic foresight relative to their younger counterparts. At the same time, age-related changes in sleep often contribute to age-related decline in other cognitive abilities known to support episodic foresight, such as memory. No study to date has tested whether sleep quality is associated with episodic foresight when it is measured behaviorally; or whether this relationship is moderated by age. To address this, in the present study healthy younger (*n* = 39) and older (*n* = 41) adults were asked to wear an actigraphy watch and self-report their sleep quality for seven nights. Participants then completed the virtual-week foresight task—a behavioral assessment of episodic foresight. Neither objective or subjective sleep quality predicted episodic foresight outcomes, and this was not moderated by age group. Bayesian analyses provided evidence in favour of the null hypotheses. These results suggest that sleep quality (at least in healthy adult populations) may not be linked to episodic foresight.

## Introduction

Episodic foresight refers to the ability to imagine possible future scenarios and use this imagining to guide future-directed behavior^1^. Due to its critical role in many complex activities of daily living (e.g., managing finances, shopping, and food preparation), episodic foresight is considered a key predictor of functional capacity and broader wellbeing^2,3^. There is now robust evidence that episodic foresight declines in late adulthood^4–7^. This comes primarily from phenomenological paradigms which show that older adults have greater difficulty engaging in the future-directed imaginative component that forms the basis for future behaviour (e.g.^4,8^). One study to date has behaviourally assessed younger and older adults’ ability to actually use these imaginings to guide future-directed behaviour^9^. This latter study revealed that older adults struggled more than younger adults not only at engaging in future-directed imaginings but in then also using those imaginings to influence future relevant behaviors^9^. This is particularly compelling evidence that age-related declines may have important functional implications.

Episodic foresight is a complex construct that imposes demands on many broader cognitive resources and neural mechanisms. In particular, episodic memory (the ability to recall and mentally re-experience specific episodes from one’s personal past) and executive control (the higher-level cognitive operations that coordinate more basic cognitive processes) are thought to be implicated in episodic foresight. This is because a central tenet of the *constructive episodic simulation hypothesis* is that simulating the future depends on the flexible retrieval and recombination of past experiences to simulate and pre-experience novel representations of future events^10^. Episodic memory is therefore thought to provide the basic building blocks for future simulations, while executive control is required to facilitate this flexible retrieval and recombination process, and simultaneously inhibit the tendency to simply recall past experiences (e.g.^10,11^). Neuroimaging studies have also found that a core network of brain regions (including the medial temporal lobe, the posterior cingulate cortex, medial prefrontal cortex, and lateral temporal and parietal regions) are activated when imagining future experiences (see^12^ for a review). These neural regions are also implicated in executive function and episodic memory^13–16^. This suggests that episodic foresight does impose demands on episodic memory and executive control.

Sleep quality is one potentially important variable that, to date, has rarely been assessed in relation to episodic foresight. This lack of empirical research is surprising given that objective and subjective indices of sleep quality have been shown to be associated with episodic memory and executive control, and to also directly impact key neural regions implicated in episodic foresight^17–23^. Meta-analyses have also demonstrated that sleep quality is consistently linked to poor performance in many cognitive domains^24–26^. Furthermore, poor subjective sleep quality has been linked to changes in the neural regions that support episodic foresight. For instance, reduced grey matter volume in lateral and medial temporal regions and the parietal cortex^27,28^, and more rapid volumetric losses in the right posterior cingulate cortex over a 2-year period^29^. Taken together, prior literature shows that sleep impacts many of the cognitive and neural processes thought to be critical to effectively engage episodic foresight.

While many variables have the potential to influence episodic foresight, sleep is of particular interest in the context of normal adult ageing. This is because not only is there some evidence that sleep might change as a function of normal adult ageing, but sleep might also be important to understanding the relationship between age and memory. For instance, a meta-analysis showed that older age is associated with objectively shorter sleep duration, sleep efficiency, REM sleep, and slow wave sleep^30,31^. More recent empirical studies have also observed that subjective sleep quality declines across the lifespan^32,33^, although see^34,35^. Notably, there is evidence suggesting that older adults may be better able to *cope* with poor sleep than younger adults. Specifically, although total sleep deprivation disrupts cognitive and affective function in both younger and older age cohorts, the magnitude of these effects are often larger for younger compared to older adults^36–38^. Indeed, a recent meta-analysis concluded that age moderates the relationship between sleep and episodic memory, such that the relationship between slow wave sleep and episodic memory is stronger in younger relative to older cohorts^39^. Such findings suggest that if sleep is related to episodic foresight, the strength of these effects may differ meaningfully as a function of age group (and specifically, may be stronger in the younger group).

Only one study to date has assessed whether differences in sleep might also be relevant to understanding episodic foresight capacities^40^, and it suggested that the nature of the relationship between sleep and episodic foresight differs quite fundamentally for younger and older adults. Specifically, in older adults, decreased sleep spindle density (a physiological marker of off-line memory consolidation) was associated with greater capacity for episodic foresight (measured via mental simulations of novel future events). In younger adults however, the reverse pattern of association was found—increased, rather than decreased, sleep spindle density was associated with a greater capacity for episodic foresight. However, for this study participants only imagined future scenarios and were not required to *use* that imagining to guide future-directed behavior. Therefore, these results cannot speak to whether sleep quality might be related to foresight capacity in a way that actually influences future behaviour. To address this gap, the current study was designed to provide the first test of the relationship between sleep quality and the behavioral application of episodic foresight in younger and older adulthood.

Finally, although a large literature now shows that sleep is associated with memory, the way sleep is measured (i.e., objectively or subjectively) appears to play a critical role in determining the magnitude of this relationship. Specifically, a recent meta-analysis showed that sleep and episodic memory associations were stronger for polysomnography-assessed sleep than self-reported sleep^39^. Indeed, some studies even suggest that subjective sleep has no relationship with prospective memory or general memory performance^41–43^. Such findings raise the possibility that any observed relationship between episodic foresight and sleep might be impacted by sleep measurement type. Thus, the final aim of this study was to assess the potential moderating role of measurement type in the relationship between sleep and episodic foresight. To this end, we measured subjective and objective sleep via self-report and actigraphy, respectively.

### The present study

This project was designed to gain a more nuanced understanding of the relationship between sleep quality and episodic foresight, and specifically whether age-related changes in sleep quality were related to age-related differences in the ability to engage episodic foresight behaviourally. We also aimed to determine if this relationship differed as a function of sleep measurement type. We predicted that there would be a relationship between sleep quality and episodic foresight, whereby poorer sleep quality would be associated with poorer episodic foresight. Further, we predicted that the relationship between sleep quality and episodic foresight would be stronger in younger relative to older adults. Finally, we predicted that actigraphy assessed sleep efficiency (i.e., objectively measured sleep) would be more strongly associated with episodic foresight than subjective sleep quality for both age groups.

## Methods

### Participants

A power calculation was conducted a priori using G*Power^20^. For the key pre-registered analyses, a minimum of 77 participants in total were required to have at least 80% power to detect a moderate effect size (*f*^2^ = 0.15) in a regression model with three predictors (age, sleep, and age $\times$ sleep interaction). Eighty-one individuals from the general community completed the study and were included in the final data analysis. This included 41 healthy older adults (*Mage* = 69.44, *SD* = 6.82, age range = 60–85; 21 female) and 39 healthy younger adults (*Mage* = 22.85, *SD* = 3.31, age range = 18–30; 20 female). To be eligible participants were required to: (1) be fluent in English; (2) have no current (or within the past 12 months) diagnosis of serious psychiatric illness (e.g., bipolar disorder), neurological disorder, neurodegenerative disorder, or sleep disorder; (3) not be taking sleep-altering medication; and (4) have no history of severe head trauma. An additional eight participants were tested but were excluded due to scoring above 5 (out of 8) on the STOP-BANG^44^ criteria for Obstructive Sleep Apnoea, and an additional three participants were tested but later excluded for reporting psychiatric illnesses, or severe sleep disturbances during testing.

All participants scored above the cut-off for abnormal cognitive functioning (> 21/30) on the Mini-Addenbrooke’s Cognitive Examination (^45^, see Table 1 for demographic results). As can be seen in Table 1, older and younger adults did not differ in social frailty, attention and task switching capacity (indexed via the trail making test), verbal fluency, sleep quality (indexed via the Pittsburgh Sleep Quality Index), sleep duration (indexed via actigraphy assessed total sleep time in minutes), or years of education. However, relative to younger adults, older adults had higher predicted full-scale IQ (indexed via the NART-II), poorer inhibitory function (indexed via the Stroop colour-word test), and higher daytime sleepiness (indexed via the Epworth Sleepiness Scale).

**Table 1:** Means, standard deviations, independent samples *t*-tests, and Bayesian *t*-tests across younger and older adults for background measures.

Measure	Young adults		Older adults		*t*	*df*	*p*	Lower 95%CI	Upper 95%CI	*d*	BF_10_
	*M*	*SD*	*M*	*SD*							
BMI	22.92	4.49	28.77	18.95	1.88	78	0.064	− 0.02	0.86	0.42	1.063
Education	15.85	2.18	15.90	3.25	0.09	78	0.928	− 0.42	0.46	0.02	0.233
NART-II errors	18.74	5.30	13.34	7.09	3.84	78	< 0.001	− 1.32	− 0.40	0.86	> 10.000
NART-II full scale IQ	112.21	4.41	116.29	5.75	3.56	78	< 0.001	0.34	1.25	0.80	> 10.000
STROOP	50.38	10.15	37.42	12.14	5.17	78	< 0.001	− 1.63	− 0.68	1.16	> 10.000
M-ACE	28.95	1.21	28.34	1.77	1.78	78	0.079	− 0.84	0.05	0.40	0.912
TMT	0.52	0.40	0.52	0.41	0.54	78	0.516	− 0.32	0.56	0.15	0.264
FASA	68.82	13.62	70.63	16.86	0.53	78	0.599	− 0.32	0.56	0.12	0.262
STOP-BANG	1.41	0.97	2.73	1.05	5.85	78	< 0.001	0.82	1.79	1.31	> 10.000
ESS	7.38	4.03	6.35	3.30	1.21	72	0.230	− 0.74	0.18	0.28	0.452
SFI	1.03	1.25	1.51	1.60	1.47	75	0.147	− 0.12	0.78	0.34	0.596
EF-Items used (uncond.)	71.79	17.80	46.69	32.89	4.22	78	< 0.001	− 1.40	− 0.48	0.94	> 10.000
PSQI global	7.54	3.95	6.66	3.33	1.07	76	0.288	− 0.69	0.20	0.24	0.386
ACTI-WASO	84.35	31.29	64.40	26.61	3.08	78	0.003	− 1.14	− 0.24	0.69	> 10.000
ACTI-TST	399.84	54.80	408.29	62.46	0.64	78	0.523	− 0.30	0.58	0.14	0.278
ACTI-SE	82.26	6.24	86.20	5.86	2.91	78	0.005	− 1.10	− 0.20	0.65	8.307
Subjective sleep quality	3.40	0.72	3.51	0.56	0.78	78	0.436	− 0.27	0.61	0.18	0.303

*d* Cohen’s d measure of effect size, *Lower/Upper 95%CI* lower and upper 95% confidence intervals, *BMI* body mass index, *Education* years of full-time education, *NART-II* National adult reading test-second edition, *M-ACE* Mini-Addenbrooke’s cognitive examination, *TMT* trail making test, *FASA* measure of verbal fluency, *STOP-BANG* measure for risk of obstructive sleep apnea, *ESS* Epworth sleepiness scale, *SFI* social frailty index, *EF-Items used (uncond.)* virtual week-foresight task – percentage of correct items used, *PSQI global* Pittsburgh Sleep Quality Index global score, *ACTI-WASO/TST/SE* actigraphy assessed wake after sleep onset/total sleep time/sleep efficiency, *BF* Bayes factor indicative of evidence for the alternate hypothesis.

### Focal measures

#### Objective sleep

Objective sleep was measured via the ActiGraph GT9X Link watch (35 mm $\times$ 35 mm $\times$ 100 mm, and 14 g). Actigraphy devices are widely used in research and have been validated against polysomnography—the gold standard objective sleep measurement^23^. The Actiwatch was configured as per the manufacturer’s recommendations using the ActiLife software version 6.13.4. The Actiwatch showed a standard watch face display (date and time), and participants were unable to adjust any watch settings. Participants continuously wore the Actiwatch on their non-dominant wrist for seven consecutive days and nights. Raw data were analysed using the Cole-Kripke sleep scoring algorithm^23^. Data coded from the Actiwatches was averaged across the seven nights to calculate a participant’s average total sleep time in minutes (ACTI-TST), sleep efficiency (ACTI-SE; calculated by dividing ACTI-TST by the total time in bed), and wake after sleep onset (ACTI-WASO). ACTI-TST assessed the amount of time spent asleep in minutes with higher scores indicating longer sleep duration (younger adult range = 278.86–540.14; older adult range = 306.75–550.29). The primary outcome of focus was ACTI-SE as this provided an objective measure of sleep quality. Higher ACTI-SE scores suggested better sleep efficiency—or a higher amount of total sleep time relative to time spent in bed (younger adult range = 67.92–95.05; older adult range 73.69–95.91). ACTI-WASO indexed the amount of wake time experienced after the onset of sleep, whereby higher scores indicated more time spent awake in minutes (younger adult range = 25.43–156.00; older adult range = 23.71–127.43).

#### Subjective sleep

During the seven-night period that the Actiwatch was recording, participants were asked to complete the Consensus Sleep Diary (CSD^24^) each morning as soon as they arose. This was a 9-item measure asking participants to write down their prior night’s bedtime, sleep duration, sleep onset latency, number of awakenings through the night, wake after sleep onset, time out of bed in the morning, quality of sleep, and any unusual occurrences that could have impacted their sleep or any time periods that the Actiwatch was removed. Data from the CSD supplemented the actigraphy data to determine participant’s objective sleep periods. To assess subjective sleep quality specifically, participants were asked to rate the quality of their prior night’s sleep on a scale from 1 (very poor) to 5 (very good). Ratings were then averaged across the week for each participant to indicate their average subjective sleep quality, whereby higher scores indicated better subjective sleep quality.

#### Episodic foresight

**Figure 1:**
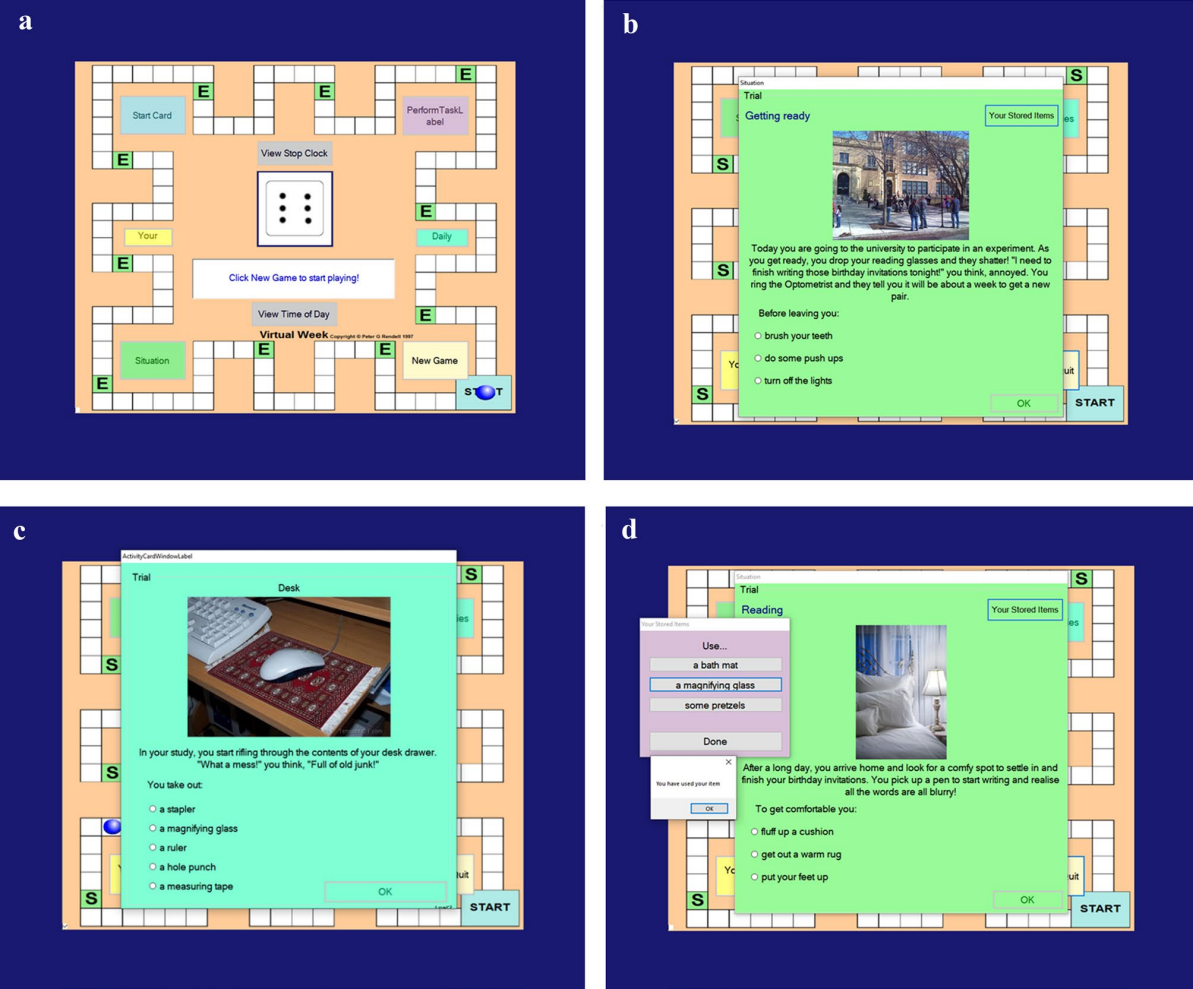
Virtual week-foresight task overview.

Virtual Week-Foresight (VW-Foresight^9^) was used to assess behavioral episodic foresight. It is a validated computerised task that is sensitive to episodic foresight difficulties associated with normal adult aging^9^. VW-foresight was presented in the form of a computerized board game. Participants were asked to use their mouse to roll a die on the screen and move their token around the board with each circuit representing one virtual day (see Fig. 1a). As participants progressed around the board, they were required to identify problems as they arose (see Fig. 1b), subsequently and spontaneously acquire an item at a later point to solve that problem (see Fig. 1c), and then return to the initial problem and solve it with the acquired item—all without any overt cueing (see Fig. 1d; see^9^, for a detailed description).

The number of correct items acquired and used across the two virtual days were then summed. The three outcome scores were: (1) *items acquired,* which reflected the number of correct items acquired expressed as a percentage of the total correct number of items that were available; (2) *items used (unconditional),* which reflected the number of correct items used expressed as a percentage of the total number of correct items that could be used; (3) *items used (conditional)*, which reflected the number of correct items used expressed as a percentage of the total number of correct items that were acquired. The first and third outcomes were the key outcomes of interest (see^9^ for a detailed explanation).

### Procedure

In the first testing session, participants read the information sheet and provided written informed consent. They then completed all background measures and received an Actiwatch and sleep diary. Participants wore the watch on their non-dominant wrist until their second testing session. During this time, participants also completed the CSD each morning once they arose. In the second testing session, participants completed the VW-Foresight task. All participants were compensated $20 per hour in the form of gift cards for their time ($60 in total for participating in the study). This study was approved by the University of Queensland Human Research Ethics Committee (Project number: HE002494). This study was conducted in accordance with relevant guidelines and regulations.

### Analyses

Analyses were conducted in JASP version 0.16.3.0. Missing data only occurred in background survey items. Specifically, data were missing from two participants in the Pittsburgh sleep quality index, from three participants in the social frailty index, and from six participants in the Epworth sleepiness scale. In these instances, analyses were conducted excluding cases per dependent variable. We first conducted a mixed-model multivariate analysis of variance (MANOVA) to assess for age differences in objective and subjective sleep quality, and age differences in episodic foresight outcomes. We then followed up significant results with independent samples *t*-tests to determine on which variable(s) age differences appeared. Bivariate correlations were conducted to assess whether sleep quality was associated with episodic foresight outcomes in younger and older adults. Four moderated regression analyses were then conducted to determine if age moderated the effect of sleep quality on episodic foresight. The moderation was also conducted with nonparametric bootstrapping to provide a robust analysis technique to account for any violations in normality or homoscedasticity. As this did not change the overall findings, results of the Bootstrapped moderations are reported in Table S1 of the Supplementary Document. Moderation analyses were conducted using the PROCESS Macro^46^ in R version 4.1.0. The JASP file and R code used are available online (see link in Data availability). For all statistical analyses, *ps* < 0.05 were considered significant.

Bayesian analyses were used to assess the strength of data in favour of the alternative versus null hypotheses. Priors were maintained as the default of equal probability of the null hypothesis and alternate hypothesis occurring. A Bayes Factor (BF) quantifies the relative predictive performance of the null hypothesis compared to the alternate hypothesis. A BF_10_ was indicative of evidence for the alternative hypothesis. Importantly, BFs are interpretable as representing a magnitude of evidence for one hypothesis over another: e.g., a BF_10_ of 3 suggests the data are three times more likely under the alternative hypothesis than under the null hypothesis. We calculated BF_10_ in JASP and as such, BF_10_s less than 1 were indicative of evidence for the null hypothesis and BF_10_s more than 1 were indicative of evidence for the alternative hypothesis. BF_10_ between 0.33 and 3 indicated weak evidence. BF_10_ between 0.1 to 0.33 or 3 to 10 indicated moderate evidence. BF_10_ less than 0.10 or more than 10 indicated strong evidence (as in Ref.^47^). Bayesian independent samples t-tests were conducted to assess the strength of any age effects. Bayesian correlations were then run to assess the strength of the relationships between key variables of interest. We then conducted Bayesian multiple regressions to determine the strength of the null effects of sleep quality in predicting episodic foresight.

## Results

### Age differences in sleep

To test for the presence of age differences in sleep quality, a MANOVA (age group was a between-subjects factor; ACTI-SE and subjective sleep quality were within-subjects factors) was conducted. We found that there was an effect of age on sleep quality *F*(1, 2) = 4.258, *p* = 0.018, *η* _p_^2^ = 0.10. Independent samples *t*-tests found no age differences in subjective sleep quality (*t* = 0.78, *p* = 0.436, 95% CI [− 0.61, 0.27], *d* = 0.18, BF_10_ = 0.303), however younger adults had significantly poorer objective sleep efficiency than older adults (*t* = 2.91, *p* = 0.005, 95% CI [− 1.10, − 0.20], *d* = 0.65, BF_10_ = 8.307; see Table 1 for means and standard deviations).

### Age differences in episodic foresight

**Figure 2:**
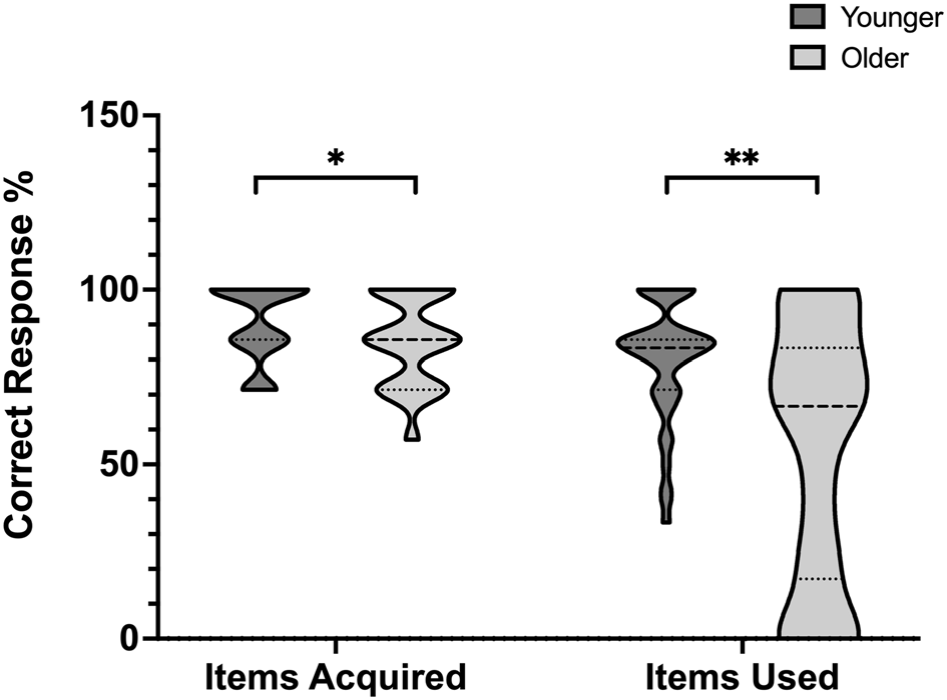
Performance on VW-foresight for younger and older participants. Items acquired and are expressed as a percentage of 7 possible items. Items used (conditionalized) are expressed as a proportion of acquired items that were used. *p < 0.05, **p < 0.01. *VW* virtual week.

To test for the presence of age differences in VW-foresight, a MANOVA (age group was a between-subjects factor; percentage of items acquired, and items used conditional were within-subject factors) was conducted. This analysis revealed a main effect of age group *F*(1, 2) = 8.573, *p* < 0.001, *η *_*p*_^2^ = 0.18. Follow-up independent samples *t*-tests found that compared to younger adults, older adults performed more poorly on both aspects of the VW-Foresight task. Specifically, older adults acquired a lower percentage of correct items (*t* = 2.04, *p* = 0.044, 95% CI [− 0.90, − 0.01], *d* = 0.46, BF_10_ = 1.391) and used a lower conditional percentage of correct items (*t* = 3.84, *p* < 0.001, 95% CI [− 1.32, − 0.40], *d* = 0.86, BF_10_ = 101.564; see Fig. 2).

### Sleep quality and episodic foresight

No significant correlations emerged between sleep quality and VW-foresight performance in either age group, irrespective of sleep measurement type (see Table 2). Moderated regression analyses revealed that sleep quality, when measured objectively or subjectively, did not predict episodic foresight capacities, and this was not moderated by age group (see Table 3).

**Table 2:** Pearson and Bayesian correlations between sleep quality and episodic foresight variables separated by age group.

Variable		1.	2.	3.	4.
1. Items acquired	Pearson’s r	–	0.018	0.154	0.218
	BF_10_	–	0.200	0.305	0.468
2. Items used (cond.)	Pearson’s r	0.159	–	0.050	− 0.044
	BF_10_	0.313	–	0.208	0.206
3. ACTI_SE	Pearson’s r	− 0.056	− 0.125	–	0.206
	BF_10_	0.206	0.261	–	0.426
4. Diary quality	Pearson’s r	− 0.188	0.022	0.050	–
	BF_10_	0.381	0.196	0.204	–

Results below the diagonal are for older adults. Results above the diagonal are for younger adults.

*BF*_*10*_ Bayes Factor indicative of evidence for the alternate hypothesis, *ACTI-SE* actigraphy assessed sleep efficiency. Items acquired = percentage of correct items acquired. Items used (cond.) = the proportion of items used to items acquired. No correlations were significant.

**Table 3:** Results of moderated regression analyses for each episodic foresight outcome.

Predictor	*B*	*SE*	*t*	*p*	Lower 95% CI	Upper 95% CI	*R*^2^	*F*	*p*	*R*^2^ change
Model 1. Items acquired as DV										
ACTI-SE	0.67	0.71	0.95	0.347	− 0.74	2.07	0.06	1.671	0.180	0.01
Age group	− 5.78	2.83	2.04	0.045	− 11.42	− 0.13				
Interaction	− 0.40	0.45	0.87	0.385	− 1.29	0.50				
Model 2. Items acquired as DV										
Diary quality	10.95	6.29	1.74	0.086	− 1.59	23.48	0.09	2.48	0.068	0.04
Age group	− 5.41	2.66	2.03	0.046	− 10.72	− 0.11				
Interaction	− 7.63	4.29	1.78	0.079	− 16.17	0.91				
Model 3. Items used (cond.) as DV										
ACTI-SE	1.07	1.73	0.62	0.537	− 2.38	4.52	0.17	5.210	0.002	0.01
Age group	− 24.01	6.96	− 3.45	< 0.001	− 37.88	− 10.14				
Interaction	− 0.94	1.11	− 0.84	0.401	− 3.14	1.27				
Model 4. Items used (cond.) as DV										
Diary quality	− 3.56	15.79	0.23	0.823	− 35.01	27.90	0.16	4.82	0.004	< 0.001
Age group	− 25.29	6.68	3.78	< 0.001	− 38.60	− 11.98				
Interaction	2.51	10.76	0.23	.816	− 18.92	23.95				

Degrees of freedom for *F* statistic = 3, 76.

*ACTI-SE* actigraphy assessed sleep efficiency. Items acquired = percentage of correct items acquired. Items used (cond.) = the proportion of items used to items acquired.

### Bayesian analyses

There was moderate evidence for the alternate hypothesis that ACTI-SE (BF_10_ = 8.307) was different for younger and older adults. There was moderate evidence for the null hypotheses that subjective sleep quality (BF_10_ = 0.303) did not differ between younger and older adults. The evidence for the null hypothesis that sleep quality does not predict percentage of items acquired was moderate for both ACTI-SE (BF_10_ = 0.240), and subjective sleep quality (BF_10_ = 0.232). Bayesian evidence for the null hypothesis that sleep quality does not predict percentage of items used (conditionalized) was moderate for subjective sleep quality (BF_10_ = 0.243), and weak for ACTI-SE (BF_10_ = 0.727).

## Discussion

The present study provided the first test of whether sleep quality (self-reported and actigraphy assessed sleep efficiency) is related to the behavioral application of episodic foresight, as well as whether any age-related changes in sleep quality might contribute to age-related deficits in episodic foresight capacity. In line with the broader literature^9^, the results showed that older adults had poorer episodic foresight relative to their younger counterparts, and that this age effect emerged in relation to both components of the virtual-week task. Specifically, older adults initially acquired fewer necessary items than younger adults, with this effect moderate-sized in magnitude, although the associated BF_10_ suggested the presence of only weak evidence for this effect. Thus, although age-differences were found in the number of items acquired, the robustness of this age effect will need to be assessed in future research. Older adults also subsequently used a lower proportion of items acquired than their younger counterparts. This latter group difference was large in magnitude (supported by a BF_10_ that was greater than 100), suggesting very strong evidence for this age effect. Contrary to our predictions, we found no age differences in subjective sleep quality, and a BF_10_ suggested moderate evidence for this null result. We also found that older adults demonstrated better actigraphy assessed sleep efficiency than younger adults and this effect was moderate (supported by a BF_10_ greater than eight). Indeed, our confidence intervals for each of these age effects suggest high precision. Importantly, and contrary to our predictions, sleep quality was not associated with episodic foresight, nor did this finding change as a function of age group. Bayesian analyses provided moderate evidence for each of these null effects. Taken together, these findings provide consistent evidence that sleep quality is unrelated to the capacity for episodic foresight, and this is true at both younger and later stages of the adult lifespan.

The finding that subjective sleep quality did not differ between younger and older adults might be regarded as surprising, however this is not the first study to have found no age effect on this measure. Indeed, one study reported no age effect in subjective sleep quality^34^, while a separate study of more than 2000 individuals found that subjective sleep quality increased as a function of adult age^35^. Moreover, although we found that objective sleep efficiency was reduced for younger relative to older adults, the average objective sleep efficiency for each age group was very much in line with the values reported in the broader literature^48–56^. It is also of note that the only meta-analysis to date to assess the relationship between age and actigraphy assessed sleep efficiency found that the relationship was small in magnitude, and that the removal of individual studies rendered the relationship nonsignificant, implying the presence of considerable inter-study variance^30^. The current findings therefore add to growing literature that suggests actigraphy assessed sleep efficiency might be a particularly heterogeneous aspect of sleep, and not one that reliably differentiates younger and older cohorts. One potential factor that seems likely to contribute to heterogeneity in age effects is also whether people at high risk of sleep disorders were permitted to contribute. In the current research design, they were not, and during the recruitment screening process, more than 10 older adults were excluded due to scoring above a high-risk threshold for obstructive sleep apnea via the STOP-BANG. Therefore, although we found an inverse age difference in sleep efficiency, these scores are not abnormal in comparison to the broader literature and may have been influenced by the fact that we included only healthy older adults who sleep well.

Moreover, our finding that subjective sleep quality was not related to episodic foresight aligns with the broader sleep and memory literature, which have typically revealed null or small effects for prospective memory and episodic memory (e.g.^39,41,42^). However, as noted earlier, there is more compelling evidence for an association between polysomnography assessed sleep and memory^39^. Our finding that objective sleep efficiency was also not related to episodic foresight ability was therefore unexpected, and as noted, contrary to predictions. Indeed, the only prior study to date to assess objective sleep and episodic foresight found that episodic foresight was linked with sleep spindle density, and that this relationship differed across younger and older adults^40^.

One possible reason for this disconnect is that there is a fundamental difference in how sleep was indexed in the prior^40^, and present study. Sleep spindles are neural oscillations that occur in short bursts and characterize the non-rapid eye movement sleep phase. They are theorized to represent memory consolidation processes^57^. Although sleep spindles have been shown to predict objective sleep quality^58^, sleep spindles and sleep efficiency are two distinct components of sleep physiology. Indeed, a recent study found sleep spindles, but not sleep efficiency, impacted reasoning ability and cognitive function^58^. Furthermore, a recent study assessing prospective memory, sleep, and age found that age, but not actigraphy assessed sleep, was associated with prospective memory performance in a lifespan sample^59^. Therefore, sleep spindles, but not sleep quality or efficiency may be a more important predictor of cognitive function, and further research is now needed to establish whether this extends to the behavioral application of episodic foresight.

Finally, while the present study had a number of important strengths, including the use of both objective and subjective measures of sleep quality, and a behavioral paradigm to behaviorally assess episodic foresight, some limitations should be noted. Although actigraphy assessed sleep is well validated^60,61^, the current gold standard measurement for sleep is via polysomnography. Therefore, it is important for future research to confirm these null effects with polysomnography. As noted, it would also be of considerable interest to establish whether (as for other cognitive domains), sleep spindles predict the ability to engage episodic foresight on a behavioral task.

## Conclusion

Consistent with prior research, this study showed that older adults are less likely to engage and apply episodic foresight, but uniquely extends this literature to show for the first time that these age-related difficulties are unrelated to their perceived and objectively indexed sleep quality. The study also shows for the first time that the behavioral application of foresight is unrelated to sleep quality in both younger and older cohorts, with these findings robust across both subjective and objective indicators. Given sleep problems have been referred to as an emerging global pandemic^62^, these data suggest that complex activities of daily living that rely on episodic foresight ability (e.g., managing finances, shopping, and food preparation) might not be impacted by day-to-day sleep disturbances in sleep quality. In addition to investigating other important sleep parameters (such as spindle density), future research should now focus on testing other potential mechanisms that might contribute to age-related decline in episodic foresight.

## Supplementary Information

Supplementary Table S1.

## Data Availability

This study was pre-registered with the Open Science Framework. The pre-registration document and de-identified data can be accessed via the following link: https://osf.io/mybng/?view_only=fe3ba8c830d240a9aa9f0266009606ce.
