# Supplementary material for: Subjective and objective sleep quality does not predict behavioural episodic foresight in younger or older adults
Source: Sci Rep. 2023 Jan 19;13:1056. doi: 10.1038/s41598-023-28183-1 (PMC9852464; doi:10.1038/s41598-023-28183-1)
Supplement: Supplementary file 1 — Supplementary Table S1. [file 41598_2023_28183_MOESM1_ESM.docx]

**Subjective and objective sleep quality does not predict behavioural episodic foresight in younger or older adults**

| Table S1  Results of Bootstrapped moderated regression analyses. | | | | | |
| --- | --- | --- | --- | --- | --- |
| Sleep measurement | B | BootMean | BootSE | BootLLCI | BootULCI |
| Model 1. Items acquired as DV | | | | | |
| ACTI-SE | 0.572 | 0.64 | 0.67 | -0.59 | 2.05 |
| Age group | -5.691 | -5.62 | 2.73 | -10.91 | -0.22 |
| Interaction | -0.300 | -0.35 | 0.39 | -1.17 | 0.36 |
| Model 2. Items acquired as DV | | | | | |
| Diary quality | 10.95 | 10.78 | 6.01 | -1.03 | 22.53 |
| Age group | -5.41 | -5.54 | 2.67 | -10.63 | -0.30 |
| Interaction | -7.63 | -7.44 | 4.02 | -15.10 | 0.89 |
| Model 3. Items used (conditional) as DV | | | | | |
| ACTI-SE | 0.71 | 0.86 | 1.14 | -1.09 | 3.29 |
| Age group | -25.01 | -24.68 | 6.33 | -37.43 | -12.61 |
| Interaction | -0.57 | -0.71 | 0.77 | -2.53 | 0.52 |
| Model 4. Items used (conditional) as DV | | | | | |
| Diary quality | -3.56 | -4.53 | 13.47 | -31.75 | 21.50 |
| Age group | -25.29 | -25.51 | 6.67 | -38.83 | -12.71 |
| Interaction | 2.51 | 3.03 | 11.33 | -18.91 | 25.50 |
| Note. The final estimate of the moderation is computed across 5000 bootstrap samples. ACTI-SE = actigraphy assessed sleep efficiency. Items acquired = percentage of correct items acquired in Episodic Foresight task. Items used (unconditional) = the proportion of items used to items acquired. | | | | | |
